# Modification of bladder thermodynamics in stress urinary incontinence patients submitted to trans-obturator tape: A retrospective study based on urodynamic assessment

**DOI:** 10.3389/fbioe.2022.912602

**Published:** 2022-08-19

**Authors:** Hui-Hsuan Lau, Cheng-Yuan Lai, Hsien-Yu Peng, Ming-Chun Hsieh, Tsung-Hsien Su, Jie-Jen Lee, Tzer-Bin Lin

**Affiliations:** ^1^ Division of Urogynecology, Department of Obstetrics and Gynecology, Mackay Memorial Hospital, Taipei, Taiwan; ^2^ Department of Nursing, Mackay Junior College of Medicine, Nursing and Management, Taipei, Taiwan; ^3^ Department of Medicine, MacKay Medical College, Taipei, Taiwan; ^4^ Institute of Biomedical Sciences, MacKay Medical College, New Taipei, Taiwan; ^5^ Department of Surgery, Mackay Memorial Hospital, Taipei, Taiwan; ^6^ Institute of New Drug Development, College of Medicine, China Medical University, Taichung, Taiwan; ^7^ Cell Physiology and Molecular Image Research Center, Wan Fang Hospital, Taipei Medical University, Taipei, Taiwan; ^8^ Department of Physiology, School of Medicine, College of Medicine, Taipei Medical University, Taipei, Taiwan

**Keywords:** trans-obturator tape (TOT), stress urinary incontinence (SUI), thermodynamics, pressure-volume (P-V) loop, urodynamics

## Abstract

**Importance:** It needs to be clarified whether trans-obturator tape (TOT)-enhanced urethral resistance could impact the voiding function.

**Objective:** Although TOT has been well-recognized for enhancing urethral resistance to restore continence in stress urinary incontinence (SUI) patients, whether the bladder’s voiding functions adapt to the TOT-enhanced resistance has not been adequately investigated. This study thereby aimed to investigate whether TOT impacts the bladder’s thermodynamic efficacy during the voiding phase.

**Design:** A retrospective analysis of urodynamics performed before and after TOT was assessed.

**Setting:** A tertiary referral hospital in Taiwan.

**Participants:** A total of 26 female SUI patients who underwent urodynamic investigations before and after TOT.

**Main outcomes and measures:** The area enclosed by the pressure-volume loop (Apv), which represents the work performed by the bladder during voiding, in a pressure-volume analysis established by plotting the detrusor pressure versus intra-vesical volume was retrospectively analyzed. Paired Student’s *t*-tests were employed to assess the difference in values before and after the operation. Significance in difference was set at *p* < 0.05.

**Results:** TOT increased Apv in 20 of 26 (77%) patients and significantly increased the mean Apv compared to the preoperative control (2.17 ± 0.18 and 1.51 ± 0.13 × 10^3^ cmH_2_O-ml, respectively *p* < 0.01). TOT also increased the mean urethral resistance (1.03 ± 0.30 vs. 0.29 ± 0.05 cmH2O-sec/ml, *p* < 0.01) and mean voiding pressure (25.87 ± 1.72 and 19.30 ± 1.98 cmH2O *p* < 0.01) but did not affect the voided volume and voiding time. Moreover, the TOT-induced Apv increment showed a moderate correlation with the changes in urethral resistance and voiding pressure (both *r* > 0.5) but no correlation with changes in voided volume or voiding time. It is remarkable that the TOT-induced urethral resistance increment showed a strong correlation with changes in voiding pressure (*r* > 0.7).

**Conclusion and Relevance:** The bladder enhances thermodynamic efficacy by adapting the voiding mechanism to increased urethral resistance caused by TOT. Further studies with higher case series and longer follow-ups should assess whether this effect could be maintained over time or expire in a functional detrusor decompensation, to define diagnostic criteria that allow therapeutic interventions aimed at its prevention during the follow-up.

**Clinical Trial Registration:** (clinicaltrials.gov), identifier (NCT05255289)

## Key points

**- **Question: Do voiding functions adapt to the TOT-enhanced resistance?
- Findings: The bladder enhances its thermodynamic efficacy by adapting itself to the increased urethral resistance caused by TOT.
**- **Meaning: The bladder performs more work during voiding because of the TOT-increased resistance.

## Introduction

Weakness in muscles and/or tissue surrounding the urethra results in stress urinary incontinence (SUI), which is characterized by involuntary urine leakage on effort, physical exertion, sneezing, or coughing (Abrams et al., 2003). SUI is a common problem affecting women’s daily life physically, socially, and hygienically(Abrams et al., 2002). Conservative treatment options, such as strengthening the pelvic floor musculature (Sahin et al., 2022) and bladder training (Kaya et al., 2015), are prescribed for SUI patients. In cases where conservative therapy has failed, a mid-urethral sling is a preferred recommended treatment option (Medina et al., 2017). In 1996, the first mid-urethral sling, tension-free vaginal tape, was launched (Ulmsten et al., 1996), and subsequently, the trans-obturator tape (TOT) was developed in 2001 (Schimpf et al., 2014). TOT exhibits a satisfactory cure rate and relatively reduced invasiveness (De Leval, 2003; Waltregny et al., 2008); therefore, it has increasingly been accepted as a treatment of choice worldwide during the last decade (Huang et al., 2018).

TOT positions a narrow band of synthetic tape under the urethra (Hsiao and Kuo, 2021), and, together with the scar tissue growing after the sling implantation, the tape adds an exogenous urethral resistance that provides urinary continence during bladder distension (Roumeguère et al., 2005; Atis et al., 2009). However, whether the bladder adapts to the TOT-enhanced urethral resistance by modifying its functions has not yet been investigated (Asıcıoglu et al., 2014; Ahn et al., 2015; Natale et al., 2019a).

In fact, to our best knowledge, there are no studies reporting outcomes on thermodynamic efficacy of the bladder after the TOT procedure. Thermodynamic efficacy has been already investigated in human physiology as the expression of the work sustained by tissues. Thus, for the heart, it has been demonstrated that the ventricular function pressure-volume analysis (PVA) is informative of its thermodynamic efficacy (Suga, 1979). Recent experimental data have shown that this principle is also applicable to the bladder, showing that by calculating the PVA during cystometry and pressure-flow studies, it is possible to assess detrusor muscle thermodynamic efficacy (Peng et al., 2020). Therefore, this study aimed to assess whether bladder thermodynamic efficacy changes after TOT in response to the increased urethral resistance, using the PVA during “urodynamic investigation.”

## Methods

### Patients

This study was reviewed and approved by the ethics committee of Mackay Memorial Hospital, Taipei, Taiwan, which waived the requirement for informed consent (20MMHIS410e, 2021/03/08) and was registered on ClinicalTrials.gov (NCT05255289). Pressure-flow studies of 30 female SUI patients (<70 years old) who underwent a TOT procedure following Delorme’s description of the obturator route (Delorme, 2001) in the Mackay Memorial Hospital were retrospectively reviewed. Patients diagnosed with pelvic organ prolapse, showing preoperative storage symptoms other than SUI, such as altered bladder sensation or increased daytime urinary frequency, and those unable to return for post-operative follow-up during the first 3 months were excluded from this study. None of these patients reported post-operative complications that could affect the result of urodynamic investigations. Four patients whose urodynamic data did not shape complete pressure-volume loops were excluded. Thus, there were a total of 26 patients in the statistical analysis.

### Pressure-flow study and pressure-volume analyses

**FIGURE 1:**
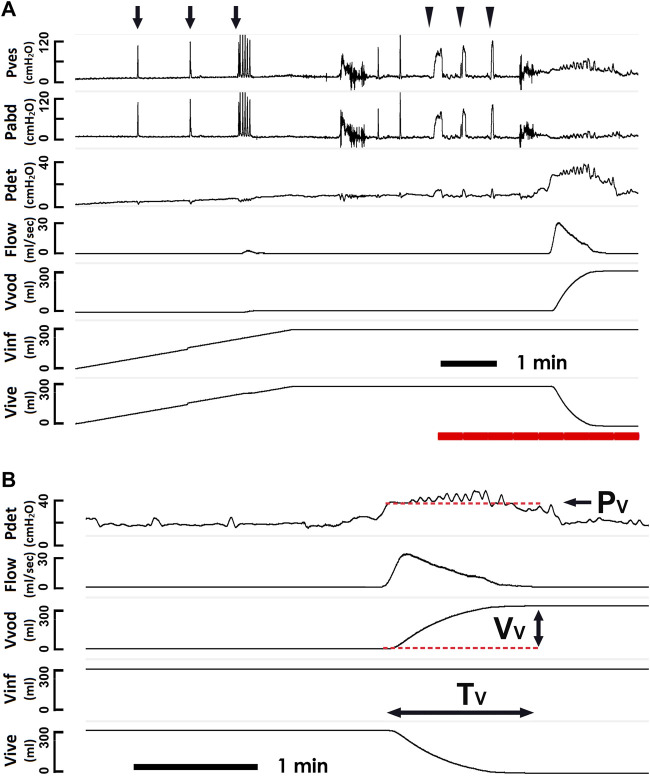
Pressure-flow study and derived voiding parameters. **(A)** representative cystometry tracings showing the vesical pressure (Pves), abdominal pressure (Pabd), detrusor pressure (Pdet), urethral flow (Flow), voided volume (Vvod), infused volume (Vinf), and intra-vesical volume (Vive). Although, coughs (arrows) and Valsalva maneuvers (triangles) induce marked fluctuations in the Pves and Pabd, they exhibit low effect on the Pdet. The Pdet, Flow, Vvod, Vinf, and Vive marked by the red bar at the bottom are showed using a faster time-base below. **(B)** derived voiding parameters including the mean voiding pressure (Pv), the voided volume (Vv), and the voiding time (Tv).

**FIGURE 2:**
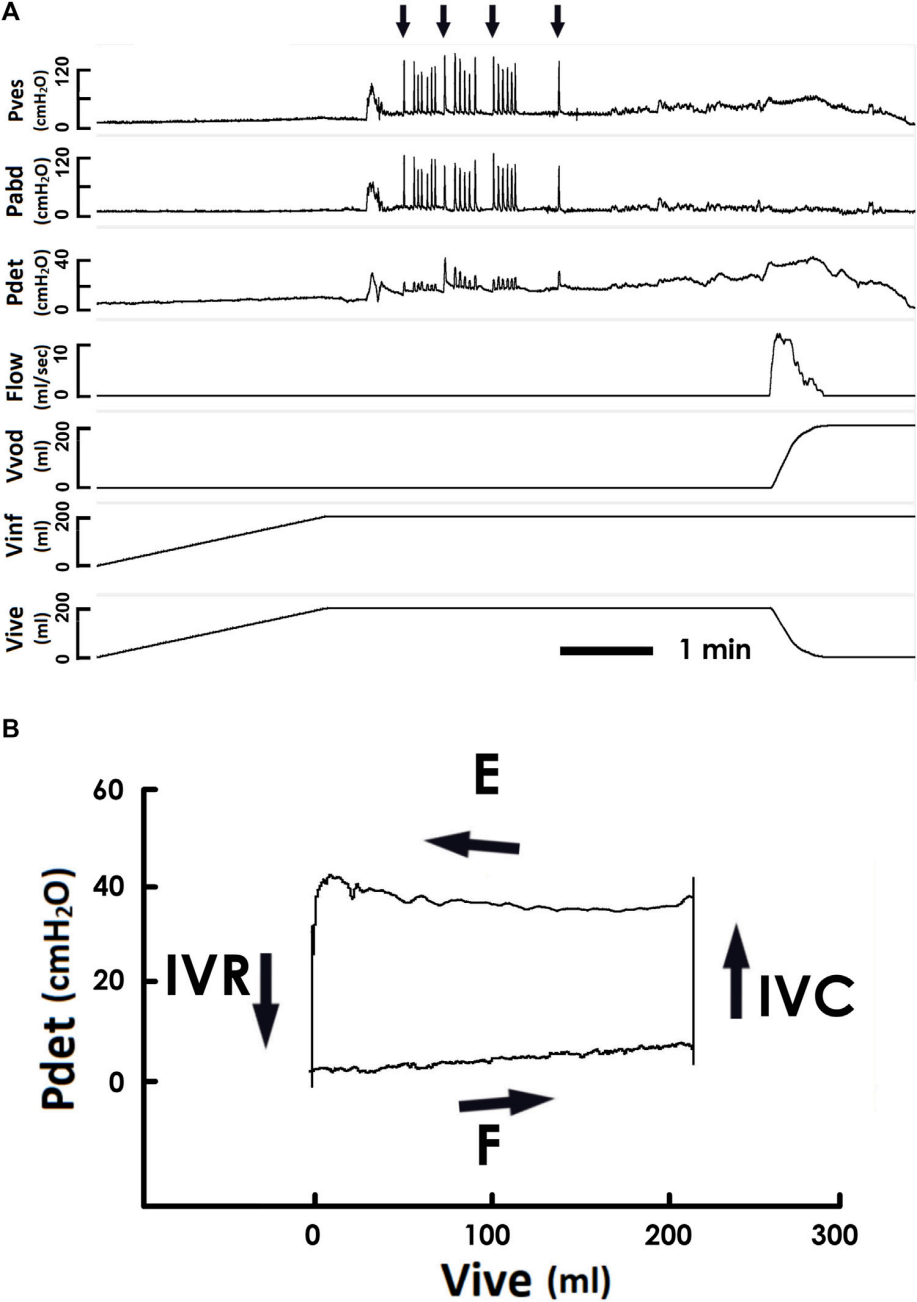
Pressure-flow/-volume analyses of a voiding. **(A)** a representative cystometry of a SUI patient. Arrows on the top indicate coughs. Pves is the vesical pressure, Pabd abdominal pressure, Pdet detrusor pressure, Flow urethral flow, Vvod voided volume, Vinf infused volume, and Vive is intra-vesical volume. **(B)** a pressure-volume analysis established by plotting the Pdet versus Vive. The trajectory of pressure-volume data moves counterclockwise and shapes an enclosed loop representing a voiding cycle. Four phases are identified in the pressure-volume loop, namely (Abrams et al., 2003) filling (F) (Abrams et al., 2002), iso-volumic contraction (IVC), (Sahin et al., 2022), emission (E), and (Kaya et al., 2015) is iso-volumic relaxation (IVR). Orthogonal projections of the top and bottom trajectories onto the abscissa represented the voided and infused volumes of a voiding cycle. Although coughs induce marked fluctuations in the vesical and abdominal pressure in the cystometry, they exhibit low influence on the pressure-volume trajectory.

Protocols of cystometry basically complied with the guidelines of the International Continence Society (ICS) (Schafer et al., 2002; D’Ancona et al., 2019), and all the cystometric studies were performed by the same person. In brief, a multi-channel urodynamic study, in which warm saline (37°C) was infused (80 ml/min) into patients’ bladders, was recorded (MMS UD-200, Medical Measurement System, Enschede, Netherlands) and analyzed (Biopac MP36, Biopac Systems, Santa Barbra, United States) using computer systems. In particular, the urodynamic data of patients were obtained from a single urodynamic session but more and repeated tests have been performed to confirm the outcomes in those with uncertain results. The vesical pressure (Figure 1A Pves), abdominal pressure (Pabd), detrusor pressure (Pdet), urethral flow (Flow), voided volume (Vvod), infused volume (Vinf), and intra-vesical volume (Vive) were recorded online, and the mean voiding pressure (Figure 1B Pv, the mean Pdet during fluid emission), the voided volume (Vv, the volume evacuated by the bladder), the voiding time (Tv, the time of fluid emission), and the mean voiding resistance (Rv, calculated by Pv/(Vv/Tv)) were analyzed off-line. Derived from the cystometry (Figure 2A), the PVA of voiding cycles was established by plotting Pdet versus Vive (Figure 2B) (Peng et al., 2020; Peng et al., 2021). Comparable to cystometry, orthogonal projection of the top trajectory onto the abscissa represented the voided volume (Vv) of a voiding cycle, and the trajectory-enclosed area (Apv) was analyzed using an image-processing program (ImageJ, LOCI, Madison, WI, United States). Although cough and Valsalva tests markedly interfered with Pves and Pabd, a previous publication (Lee et al., 2021) and our data (Figure 1A) demonstrated that these tests showed little effect on the Pdet in the cystometry. Moreover, our data discovered that cough and Valsalva maneuvers did not significantly affect the PVA trajectory. In addition, cough and Valsalva tests were carried out during bladder filling without effects on voiding dynamics. Therefore, cystometry data of SUI patients who underwent stress tests (coughs and/or Valsalva) were pooled together with those without test(s).

### Statistical analyses

All data in this study were expressed as the mean ± SEM. The difference in values between groups was assessed using paired Student’s *t*-tests. Significance in difference was set at *p* < 0.05.

## Results

### Database of patients

The 26 female patients eligible for the study were aged 54.59 ± 1.27 years. Urodynamic evaluations were respectively carried out at a mean of 52.84 ± 20.29 days before and 177.80 ± 23.26 days after the TOT surgery. No patients reported voiding symptoms before being submitted to the post-operative urodynamic assessment.

### Pressure-flow/volume analyses

In the cystometry, bladder filling was carried on until patients reported a desire to void (Figure 2A). The PVA, demonstrated the trajectory of pressure-volume data moved counterclockwise and shaped an enclosed loop representing a voiding cycle (Figure 2B). The following four phases were identified in a loop (Abrams et al., 2003): filling (F, from the beginning of filling cystometry to the voiding)—an increasing Vive together with a slightly but progressively elevated baseline Pdet (Abrams et al., 2002); isovolumic contraction (IVC, from the voiding contraction onset to the beginning of the urine emission)—the bladder contracted without fluid expulsion, which resulted in an abruptly elevated Pdet with a nearly constant Vive (Sahin et al., 2022); emission (E, from the beginning to the end of fluid emission)—a plateau Pdet that was slightly elevated at the end of this stage with a marked Vive decrease (Kaya et al., 2015); and isovolumic relaxation (IVR, from the end of emission to the end of bladder relaxation)—the bladder relaxed without fluid expulsion that resulted in a marked Pdet decline with an almost constant Vive.

### Trans-obturator tape increases the loop-enclosed area

**FIGURE 3:**
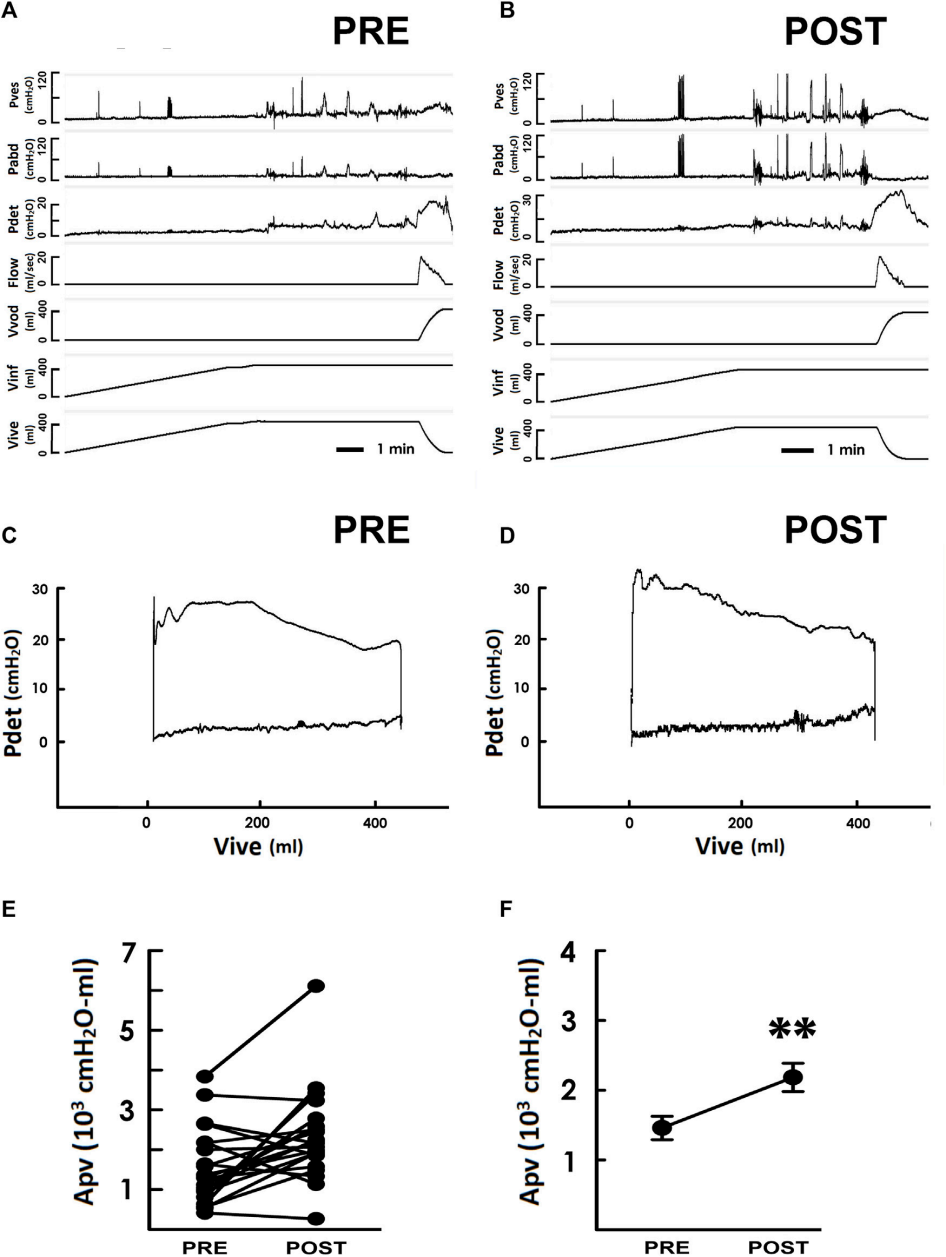
Pressure-flow/-volume analyses in response to the TOT surgery. **(A,B)** representative cystometry of a SUI patient measured pre- and postoperatively (PRE and POST, respectively). Pves is the vesical pressure, Pabd abdominal pressure, Pdet detrusor pressure, Flow urethral flow, Vvod voided volume, Vinf infused volume, and Vive is intra-vesical volume. **(C,D)** Pressure-volume analyses measured before and after the TOT surgery, respectively. **(E)** the loop-enclosed area (Apv) of each SUI patient measured pre- and post-operatively. **(F)** summarized Apv of SUI patients before and after the TOT surgery (**p < 0.01 vs. PRE; *N* = 26).

The potential impact of TOT on the thermodynamic efficacy of bladder voiding was investigated by comparing the area enclosed by the trajectory (Apv) in the pre- and post-operative PVA that represents the work of a voiding cycle (Peng et al., 2020). The PVA loops derived from the pre- (Figure 3A PRE) and post-operative (Figure 3B POST) pressure-flow data revealed an elevation of the upper border of the loop as the result of an increased Apv with respect to the preoperative value (Figures 3C,D). The TOT-increased Apv was confirmed by the summarized data in most patients (20/26, 77%, Figure 3E), and it significantly increased the mean value of Apv compared to the preoperative control (Figure 3F **p < 0.01 vs. PRE; *N* = 26).

### Trans-obturator tape increases voiding resistance

**FIGURE 4:**
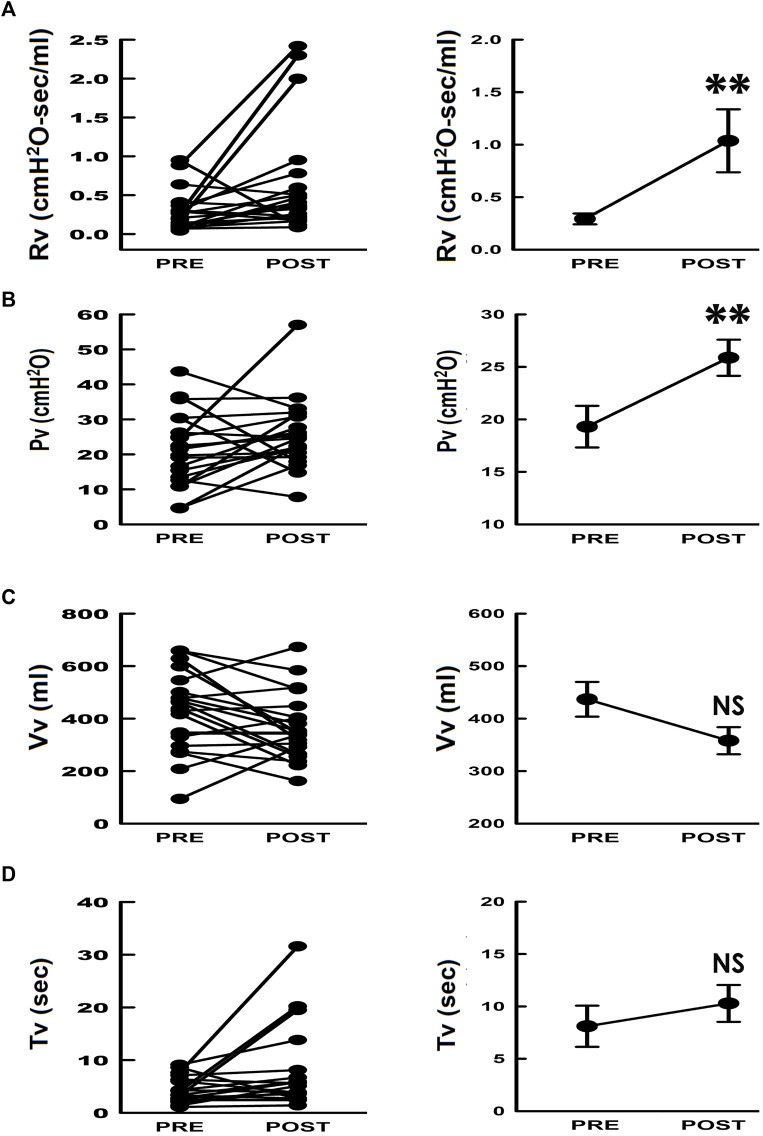
Individual and summarized data of voiding parameters in response to TOT surgery. **(A–D)** individual (left) and summarized (right) data of the mean voiding resistance (Rv), mean voiding pressure (Pv), voided volume (Vv), and voiding time (Tv), respectively. (**p < 0.01, NS *p* > 0.05 vs. PRE; *N* = 26).

TOT increased the individual mean urethral resistance (Rv) in 21 out of 26 patients (80%) and significantly increased the mean Rv value when compared to the preoperative result, suggesting an increased urethral voiding resistance induced by TOT. Furthermore, TOT increased individual mean urethral resistance (Rv) in most patients (21/26, 80%, Figure 4A) and significantly increased the mean value of Rv when compared with the preoperative control (**p < 0.01 vs. PRE; *N* = 26), indicating TOT did increase the outlet resistance during voiding.

### Trans-obturator tape increases voiding pressure

The mean resistance during voiding was defined as the mean voiding pressure (Pv) divided by the mean urethral flow (Fv, Rv = Pv/Fv), and the Fv could be further calculated by dividing the mean voided volume (Vv) by the voiding time (Tv, Fv = Vv/Tv). The relationship between the mean urethral resistance and these parameters could be described as Rv = Pv/[Vv/Tv]. We hence further assessed the changes in Pv, Vv, and Tv in response to surgery to specify the TOT impact. Compared with preoperative controls (Figure 4B PRE), TOT postoperatively increased individual Pv in most patients (POST, 21/26, 80%) and significantly increased the mean value of Pv (**p < 0.01 vs. PRE; *N* = 26). In contrast, individual data demonstrated neither Vv (Figure 4C, 9/26, 34% increased, 16/26, 62% decreased, 1/26, 4% unchanged) nor Tv (Figure 4D, 8/26, 31% increased, 17/26, 65% decreased, 1/26, 4% unchanged) showed a significant change (>75%). Moreover, no statistical significance was found in the mean values of Vv and Tv (NS *p* > 0.05 vs. PRE; both *N* = 26). These results indicated that added to an increased outlet resistance, TOT postoperatively elevated bladder pressure during voiding.

### Correlations between pressure-volume loop increase with voiding pressure and voiding resistance

**FIGURE 5:**
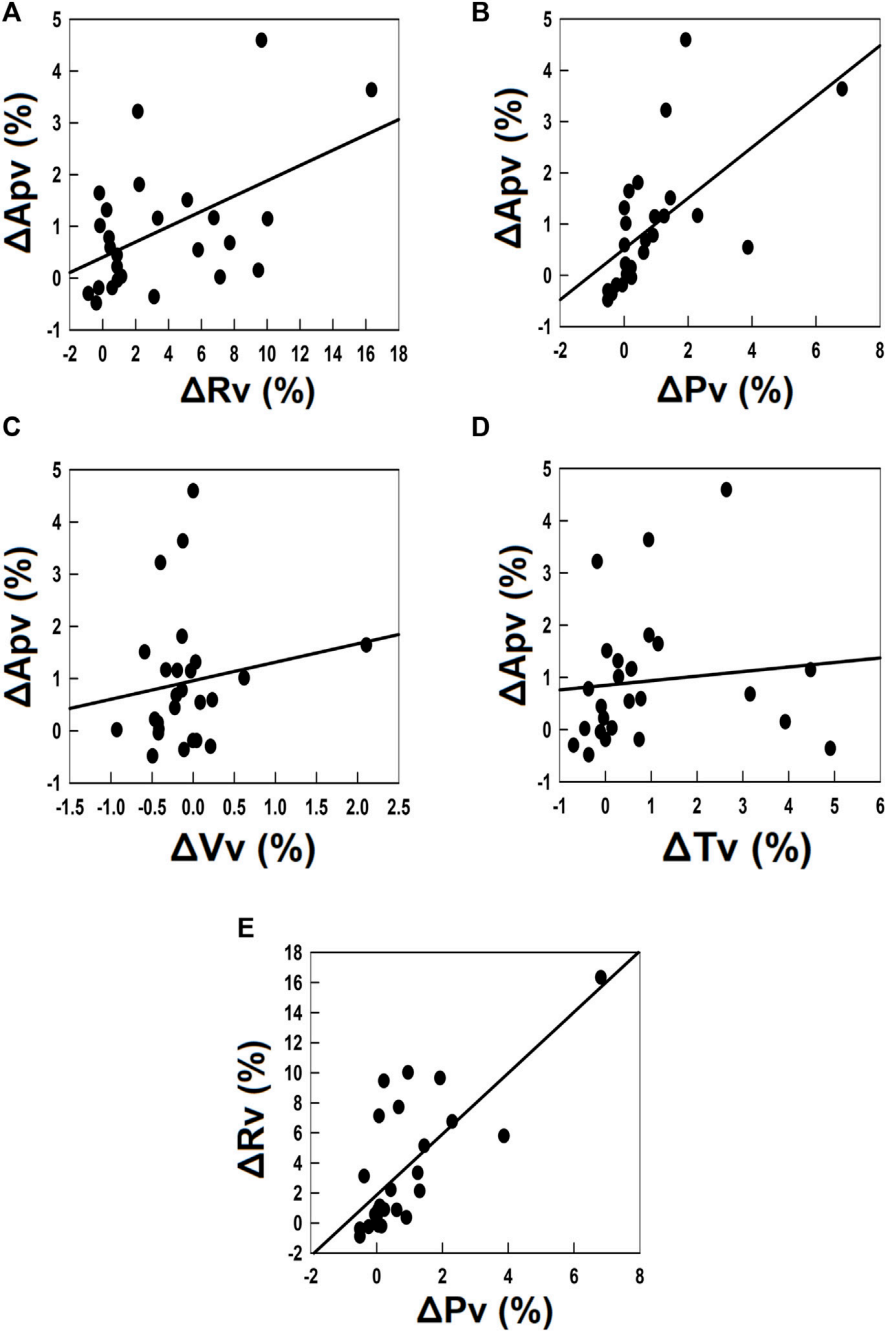
Correlation analyses of the TOT-associated change in Apv and changes in voiding parameters. **(A–D)** correlation analyses of the TOT-associated change in the loop-enclosed area (ΔApv) and changes in the mean voiding resistance (ΔRv; *r* > 0.5, *N* = 26), the mean voiding pressure (ΔPv; *r* > 0.5, *N* = 26), the voided volume (ΔVv; *r* < 0.3, *N* = 26), and the voiding time (ΔTv; *r* < 0.3, *N* = 26), respectively. **(E)** correlation analysis of ΔRv and ΔPv (*r* > 0.7, *N* = 26).

We next assessed the relationship between the TOT-enhanced Apv and changes in urodynamic parameters by analyzing the correlation between the change in Apv (ΔApv) and those in Rv, Pv, Vv, and Tv (ΔRv, ΔPv, ΔVv, and ΔTv, respectively). TOT-induced ΔApv showed moderate correlations with the ΔRv and ΔPv (Figures 5A,B. Both *r* > 0.5, *N* = 26). Nevertheless, no correlation was evidenced between the ΔApv and ΔVv or ΔTv (Figures 5C,D Both *r* < 0.3, *N* = 26). Moreover, the potential association between the TOT-induced ΔRv and ΔPv was also assessed, and the ΔRv showed a strong correlation with the ΔPv (Figure 5E *r* > 0.7 *N* = 26). Together, these results indicate the TOT-enhanced Apv was associated with a syncytial change of Rv and Pv.

## Discussion

Data collected provide evidence that the bladder increases its thermodynamic efficiency after the TOT procedure by adapting the voiding function to higher urethral resistance. This outcome is supported by the post-operative increase in Rv during the voiding phase, associated with an increase in Pv.

Our conclusion is based on lines of evidence. First, consistent with studies showing that TOT adds to outlet resistance (Sander et al., 2002) to restore adequate continence (Rapoport et al., 2007; Atis et al., 2009), we observed that TOT increased Rv during voiding. Moreover, without affecting Vv or Tv, TOT postoperatively elevated Pv. Considering that the relationship between the above parameters could be described as Rv = Pv/[Vv/Tv], unchanged Vv and Tv indicate that the TOT-increased Rv is associated with a corresponding Pv elevation. As it has been well accepted that TOT increases the outlet resistance (Rapoport et al., 2007; Atis et al., 2009), these data imply that the bladder develops an elevated voiding pressure in response to the TOT-increased resistance. Moreover, the TOT-increased Apv was correlated with ΔRv and ΔPv but not ΔVv or ΔTv, indicating that the TOT-enhanced Apv is ΔRv- and ΔPv-dependent. In addition to the evidence that the TOT-induced ΔRv is highly correlated with the ΔPv, these findings imply that TOT-enhanced Apv results from the ΔRv-associated ΔPv. Therefore, the enhanced thermodynamic efficacy of the bladder during the voiding phase is the consequence of increased pressure in response to the TOT-increased resistance. This outcome is further supported by the PVA, showing that instead of the left, right, or bottom boundaries, the upper border of the loop trajectory, which represents the pressure during the emission, was markedly shifted upward, and thereby increased the Apv. Considering that Pv was the mean bladder pressure during urine emission, these results imply that the increased Apv is attributable to an elevated Pv. As a whole, the reported data support the evidence that after the TOT procedure, the bladder develops an elevated pressure during the voiding phase to overcome the enhanced outlet resistance, as a result of an increased Apv. Our results imply that the bladder is capable of adapting itself to overcome TOT-enhanced outlet resistance. Our study further supports the efficacy of TOT in restoring urinary continence (Lebret et al., 2001) but provides the additional new information that the bladder function adapts to the increased urethral resistance caused by the tape.

We therefore suggest that prior to TOT, the bladder of SUI patients did not develop a sufficient pressure gradient not only because the pressure increase results in urine leakage during storage, but also because it possibly causes premature urine flow that brings about an early pressure decline during voiding (Gray et al., 2017). However, not limited to storage, TOT-increased outlet resistance also prevents urine leakage during early voiding, which allows the bladder to develop an acceptable pressure gradient, thereby ensuring an adequate driving force for urine flow during the whole voiding period. That is, the TOT-increased urethral resistance is of clinical benefit to both the continence and voiding functions. Although the involved mechanisms need further investigation, two candidate mechanisms could underlie the modifications that occur postoperatively in the bladder. First, below the failure threshold, the detrusor adapts itself to an inotropic situation following an enhanced outlet resistance (Elmissiry et al., 2014). As an alternative, the bladder inherently develops a higher tension to the enhanced outlet resistance because its length-tension relationship is distinctive and relatively linear (Southern et al., 2012). Nevertheless, studies investigating the adaptation of bladder contractility in response to TOT are required to clarify the precise mechanism involved. Moreover, if TOT should be avoided in bladders unable to balance the increased urethral resistance is a question that still needs to be investigated.

**FIGURE 6:**
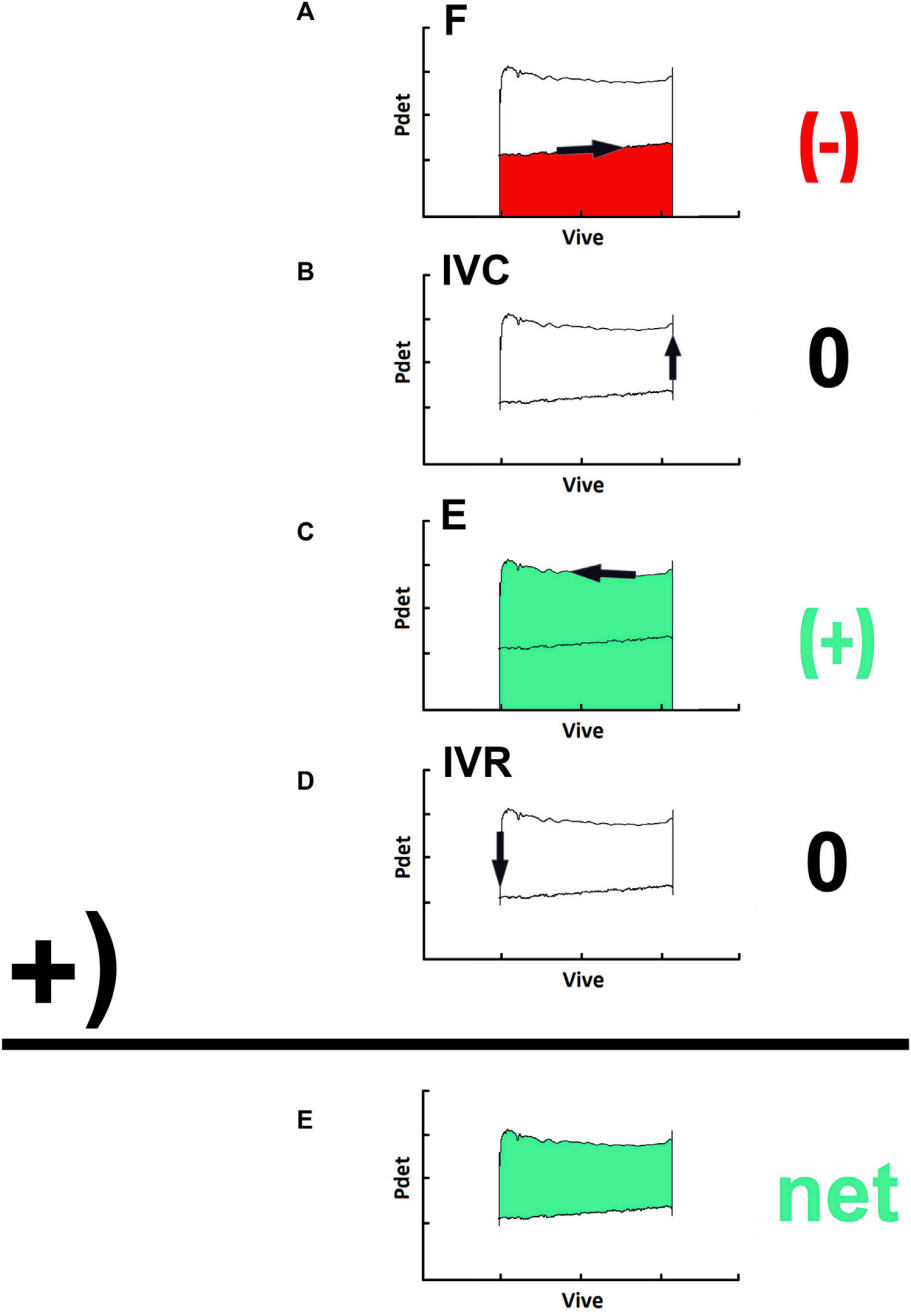
Thermodynamic processes in a pressure-volume loop of a voiding. **(A)** during the filling (F), an amount of potential energy represented by the area under the trajectory along the volume change is stored, that is, the output work is negative (−; red). **(B)** in the isovolumic contraction (IVC), the bladder performs no mechanical work, that is, the output work is zero (0). **(C)** in the emission **(E)**, the bladder performs a mechanical work characterized by the integration of the trajectory along this stage to repulse fluid, that is, the output work is positive (+; green). **(D)** in the isovolumic relaxation (IVR), the bladder gains no mechanical work, that is, the work output is zero (0). **(E)** the net work done in the entire cyclic process is given by the area enclosed by the loop trajectory of a voiding cycle (net; green).

Our study first provided the outcomes of PVA of human bladder voiding. Stepwise thermodynamic analysis revealed that during filling (F, Figure 6A), the accumulating fluid gradually increased the bladder volume with a slightly but progressively elevated baseline pressure. Thereby an amount of potential energy represented by the area under the trajectory is stored in the bladder (Peng et al., 2020). Next, in the IVC (Figure 6B), the bladder contracts without fluid elimination. Considering that there is no detrusor shortening, the bladder performs no mechanical work. Instead, it gains potential energy caused by contraction-increased stiffness. In the subsequent emission (E, Figure 6C), the bladder performs mechanical work characterized by integrating the trajectory to repulse fluid (Peng et al., 2020). At last, the bladder isovolumically relaxes without fluid elimination (IVR, Figure 6D). Considering that the detrusor length remains unchanged, the bladder gains no mechanical work, but its potential energy is reduced by the relaxation-decreased stiffness. Although this model obviously neglects the kinetic and friction energy of fluid, we suggest the Apv coarsely quantifies a simplified but neat mechanical work performed by the bladder in a voiding cycle (net, Figure 6E) (Peng et al., 2020).

Although almost all pressure/volume events in PVA can be measured as well or even better by the wide cystometry (Gammie et al., 2016; Malde et al., 2017), PVA provides a protocol for conceptually assessing the workload of voiding contractions that cannot be immediately visualized in cystometry. In association with time-domain cystometry, an additional PVA would benefit clinicians because lasting increased workload is a risk factor causing noncompensatory bladder functions (Schneider et al., 2021). Further PVA studies would provide data for stratifying PVA outcomes to establish a cut-off workload that implies a risk of detrusor decompensation. This is clinically significant because it could suggest the need for treatment to improve bladder voiding following TOT. The outcomes of our study support further research on PVA application in lower urinary tract dysfunction and disorders, such as neurogenic bladder, or inflammatory conditions (i.e., ketamine induced cystitis). In addition, it would be rather interesting to investigate further whether PVA could be used to further assay other bladder functions like it does in cardiology.

To clarify the specific urodynamic effects of urological/gynecological surgeries is clinically important because patients could develop postoperatively functional disorders that negatively impact their quality of life (Natale et al., 2019b). Although there are arguments that a preoperative urodynamic study is an unnecessary procedure or is informative about bladder function (Serati and Agrò, 2016; Serati et al., 2019), a 269-day follow-up study concluded that preoperative urodynamic examination does not affect the TOT outcomes (da Cruz et al., 2021). We thereby suggest that our preoperative urodynamic investigations could have a small effect on the TOT therapeutic effects. Moreover, considering that clinical trials demonstrated that preoperative measurements provide information on voiding/storage functions that can be compared with the post-operative data (Serati et al., 2020; Chiang et al., 2021), a preoperative urodynamic investigation is valued for objectively assessing therapeutic benefit and predicting patients requiring further therapy. In addition, considering that our results reveal that TOT shows therapeutic benefits to voiding functions of SUI patients, it would be interesting to clarify whether protocols other than TOT, particularly a retropubic sling, could exhibit similar benefits because the mid-urethral sling is recognized as a gold standard for the surgical treatment of female SUI patients (Bailly and Carlson, 2017). Based on our results, we can speculate that the positive effect on the thermodynamic efficacy of the bladder provided by the TOT prevents functional deterioration of the detrusor that would occur if incontinence persisted. In fact, urinary incontinence, especially in case of severe condition, would cause such a reduction of detrusor thermodynamic efficacy because of the absence of micturition (ex non usu), leading to a serious de-functionalization that could be irrecoverable if not managed properly.

This study has inherent limitations because of its retrospective design, and the sample size is relatively small. In addition, considering that some studies have demonstrated that some patients experience voiding dysfunctions at 12 months after TOT (Asıcıoglu et al., 2014) and that the continence rate decreases until 25 months after TOT (Costantini et al., 2016), a longer follow-up period is needed to confirm the lasting benefits of TOT because our outcomes were measured at 177.80 ± 23.26 days after the procedure.

Moreover, although our data demonstrate that TOT enhanced the outlet resistance, a very recent study showed that force vectors are responsible for the pressures and flows that develop during micturition (Ishii et al., 2020). Therefore, future studies assessing the impact of TOT using imaging techniques such as vector projectile imaging (Ishii et al., 2020) or videourodynamic imaging (Yu and Kuo, 2022) are warranted to provide clear information about the outcome of surgeries. It is worth noting that, in addition to the elevated urethral resistance, an unrelaxed perineal muscle could also result in results similar to ours. Considering that one publication demonstrates a technique of urodynamic investigation with simultaneous perineal electromyography (Talavera et al., 2022), studies investigating pressure/volume profiles using electromyograms would clarify the potential involvement of perineal relaxation in the TOT-associated impact on urodynamic variables.

## Conclusion

The results of this study demonstrate for the first time that bladder thermodynamic efficacy improves after the TOT procedure. This evidence, based on the PVA of the pressure/flow studies of the urodynamic examination, confirms the efficacy of TOT in the treatment of SUI, but it also demonstrates a preventive role against a detrusor decompensation that would inevitably occur if the incontinence persisted for a long time without any treatment.

## Data Availability

The raw data supporting the conclusion of this article will be made available by the authors, without undue reservation.
